# Socio-Environmental Determinants and Health Effects of Water Contamination Among Children in Pakistan: A Narrative Review

**DOI:** 10.7759/cureus.113778

**Published:** 2026-08-01

**Authors:** Eesha R Alladin, Rabiah Asif, Saad Karamat

**Affiliations:** 1 Public Health, Binghamton University, Binghamton, USA; 2 Public Health, Drexel Dornsife School of Public Health, Philadelphia, USA; 3 Urgent Care, Patient First, Stafford, USA

**Keywords:** arsenic exposure, child health, drinking water, groundwater contamination, lead exposure, pakistan, waterborne disease, water contamination, water insecurity, water quality

## Abstract

Pakistan is experiencing one of the world's most severe water crises, with environmental and socio-environmental factors collectively contributing to unsafe drinking water and adverse child health outcomes. Environmental determinants, including climate change, agricultural practices, and groundwater depletion, drive water contamination, resulting in exposure to toxic contaminants such as arsenic, lead, and other heavy metals. Simultaneously, socio-environmental determinants, including education, sanitation, and governance, influence children's exposure and vulnerability to contaminated water. Because children are particularly susceptible to infectious diseases, malnutrition, and neurodevelopmental injury from environmental toxicants, understanding these interconnected pathways is essential for developing effective public health strategies. This narrative review synthesizes current evidence on the environmental and socio-environmental determinants of water contamination in Pakistan and examines their relationship with child health outcomes. The available evidence indicates that protecting child health requires integrated, long-term strategies that address both the environmental drivers of water contamination and the social factors that influence children's exposure and vulnerability.

## Introduction and background

Water is essential for sustaining life, and access to a safe, adequate, and reliable water supply is fundamental to public health. The World Health Organization (WHO) emphasizes that drinking-water regulations must be adapted to each country’s specific needs, resources, and institutional capacity [1]. Pakistan ranked 31st globally for baseline water stress in the World Resources Institute’s 2023 Aqueduct 4.0 country rankings [2]. Its per capita water availability declined from approximately 1,500 m³ in 2009 to 1,017 m³ in 2021, illustrating the growing pressure on national water resources [3].

Pakistan faces growing water scarcity along with serious concerns about water quality [4,5]. Major sources of groundwater contamination include agricultural chemicals, industrial effluents, and sewage. These problems are compounded by weak enforcement and inadequate water and sanitation infrastructure [4]. Limited hygiene awareness and poor access to safe water and sanitation further increase health risks, particularly among children [6]. In the 2023 WHO/UNICEF (the United Nations Children's Fund) Joint Monitoring Programme report, Pakistan fell within the 51%-75% coverage category for safely managed drinking-water services in 2022 [7].

Children are especially vulnerable to illnesses associated with contaminated drinking water [8,9]. Diarrheal disease remains a major contributor to childhood illness and mortality in Pakistan [8,9]. Among rural school-aged children in Lahore and Kasur, lower IQ scores were more frequent in the group exposed to arsenic and fluoride through drinking water than in the control group [10]. Lead exposure has also been associated with lower IQ scores in children, with the magnitude of the reduction varying by exposure duration [11]. A review of lead exposure among children in Pakistan noted that chronic lead exposure can cause neurotoxicity and altered cognitive function [12].

Climate change is placing further pressure on Pakistan’s water resources [5,13]. Flooding can damage water systems and leave affected communities dependent on contaminated water from ponds and wells [14]. Conversely, drought reduces water availability and can increase contaminant concentrations [13]. As Pakistan depends heavily on monsoon rainfall, groundwater, and the Indus Basin for domestic water and agriculture, changing weather patterns threaten both water and food security [5,13].

This review uses a socio-environmental determinants framework to organize these interconnected pathways. Within this framework, environmental processes, including climate change, flooding, groundwater depletion, and agricultural practices, contribute to water contamination. Major contaminants and pathogens then create adverse health effects. Meanwhile, socio-environmental determinants such as education, sanitation, and governance influence the degree of children’s exposure, vulnerability, and capacity to avoid or recover from water-related illness. These interacting pathways ultimately shape infectious, nutritional, and neurodevelopmental health outcomes.

Although previous studies have examined individual aspects of Pakistan’s water crisis, including climate change, groundwater contamination, sanitation, and toxic exposures, few reviews have integrated these domains within a unified framework focused specifically on child health. This narrative review addresses that gap by synthesizing evidence across environmental drivers, major water contaminants, socio-environmental determinants of exposure and vulnerability, and associated pediatric health outcomes.

The objective of this narrative review is to examine how environmental and socio-environmental determinants contribute to water contamination and influence child health outcomes in Pakistan. Specifically​​​​​​, this narrative review examines the following research question: How do environmental drivers of water contamination and socio-environmental factors interact to influence children’s exposure, vulnerability, and health outcomes in Pakistan?

This work was previously presented as a poster presentation at the American Public Health Association (APHA) Annual Convention on November 13, 2023.

## Review

Review Methodology

This study was conducted as a narrative review examining the environmental and socio-environmental determinants contributing to water contamination and associated child health outcomes in Pakistan.

A socio-environmental determinants framework was used to organize the literature. Within this framework, environmental determinants, including climate change, agricultural practices, groundwater depletion, and toxic contaminants, were examined alongside socio-environmental determinants such as sanitation, education, and governance to evaluate how these interconnected factors influence children's exposure, vulnerability, and health outcomes (Table 1).

**Table 1:** Summary of the conceptual framework used in this narrative review Credit: This table was created by the authors of this study.

Framework Component​	Key Factors​	Effect on Water Contamination/Exposure​	Child Health Outcomes​
Environmental determinants​	(1) Climate change and flooding; (2)​ agricultural practices ​	(1) Damages water infrastructure, contaminates wells and surface water, increases pathogen exposure;​ (2) groundwater depletion, salinization, agricultural runoff.	(1) Waterborne diseases, malnutrition;​ (2) reduced water quality, food insecurity​
Major water contaminants​	(1) Lead exposure;​ (2) arsenic exposure; (3) other heavy metals​	(1) Industrial sources, aging infrastructure; (2) naturally occurring and anthropogenic contamination;​ (3) limited research on the effects of cadmium, chromium, and nickel ​	(1) IQ reduction, behavioral impairment;​ (2) growth impairment, neurodevelopmental deficits​; (3) potential chronic toxicity​
Socio-environmental determinants​	(1) Education; (2)​ sanitation;​ (3) governance​	(1) Improves hygiene practices and health-seeking behaviors; (2) reduces fecal contamination of drinking water; (3)​ water regulation, disaster preparedness, policy implementation​	(1) Reduced exposure and vulnerability​; (2) lower diarrheal disease burden; (3)​ improves water security and public health capacity​
Child health outcomes​	(1) Waterborne diseases; (2) malnutrition and growth impairment; (3) neurodevelopment​	(1) Exposure to contaminated drinking water and poor sanitation; (2)​ recurrent infections, food insecurity, arsenic exposure; (3)​ chronic lead and arsenic exposure​	(1) Diarrhea, typhoid, hepatitis, parasitic infections;​ (2) stunting, wasting, impaired growth; (3)​ lower IQ, cognitive impairment, behavioral changes​
Intervention strategies​	(1) Water governance; (2) sustainable water management;​ (3) integrated water, sanitation, and hygiene (WASH) interventions​	(1) Improved policy implementation;​ (2) groundwater regulation, drip irrigation, and reservoirs​; (3) hygiene promotion and sanitation improvements​	(1) Long-term water security​; (2) reduced contamination risk;​ (3) reduced childhood exposure and diarrheal disease​

Literature searches were conducted using PubMed and Google Scholar. The inclusion criteria consisted of articles and reports between January 2000 and May 2026 that examined water contamination, its environmental or socio-environmental determinants, or associated child health outcomes in Pakistan. Pakistan-specific studies involving children younger than 18 years were prioritized. International studies and reports were also included when they provided established evidence on the health effects of relevant contaminants or addressed areas where Pakistan-specific pediatric evidence was limited. Sources published outside the selected period, unrelated to the review objectives, or lacking relevance to water contamination or child health were excluded.

Representative search terms included Pakistan, water contamination, drinking water, water quality, water insecurity, child health, waterborne disease, arsenic exposure, lead exposure, groundwater contamination, heavy metals, sanitation, integrated water, sanitation, and hygiene (WASH), climate change, agriculture, food security, public health, and socio-environmental determinants.

Titles and abstracts of retrieved publications were screened for relevance to the review objectives, and full-text articles were reviewed for inclusion when they addressed environmental determinants of water contamination, socio-environmental determinants of exposure and vulnerability, or child health outcomes in Pakistan. Priority was given to peer-reviewed journal articles and reports from established international public health and development organizations that provided national or region-specific evidence relevant to the review objectives.

As this study was designed as a narrative review, no formal systematic review protocol, PRISMA reporting framework, quantitative meta-analysis, or formal risk-of-bias assessment was performed. Instead, evidence was synthesized qualitatively using the socio-environmental determinants framework to identify relationships among environmental drivers, water contamination, exposure and vulnerability, and pediatric health outcomes.

Environmental determinants of water contamination

Climate Change and Flooding

Although developing countries contribute relatively little to global greenhouse gas emissions, their poorest populations often face some of the greatest climate-related risks [15]. Pakistan, for example, contributes less than 1% of global greenhouse gas emissions but remains highly vulnerable to the effects of climate change [16]. Pakistan relies heavily on monsoon rainfall and glacier-fed rivers, so changes in weather patterns, including droughts and floods, can threaten its water supply and food production [5].

The 2025 floods show how vulnerable Pakistan is to extreme weather. During the summer, heavy monsoon rainfall caused widespread flooding across northern Pakistan, with Punjab and Khyber Pakhtunkhwa among the hardest-hit provinces. According to National Disaster Management Authority (NDMA) data cited by World Weather Attribution, 300 people had died by August 3, including 140 children [17]. UNICEF had previously reported that 85 children died between June 26 and July 17. Of these, 22 died in Punjab during the preceding 24 hours, mostly when their homes collapsed under heavy rainfall [18].

Agriculture Practices and Groundwater Depletion

Agriculture plays a major role in Pakistan’s economy and food supply and depends heavily on irrigation from the Indus Basin [19,20]. Around 1.2 million private tube wells extract approximately 60 billion m³ of groundwater each year, and about 85% of these wells are in Punjab. This groundwater helps farmers maintain crop production, but heavy pumping has lowered water tables, increased pumping costs, and reduced groundwater quality [20].

Poor-quality groundwater also affects the soil. In some areas, farmers irrigate their fields with saline groundwater, which adds more salt to the land. About 21% of Pakistan’s irrigated farmland is affected by some degree of salinity, placing further pressure on agricultural production [20].

Groundwater quantity and quality are closely connected. As water tables fall, farmers may have to pump from deeper or poorer-quality sources. Some irrigated areas have reported groundwater containing elevated levels of salts, fluoride, and arsenic. Together, groundwater depletion and soil salinity threaten Pakistan’s farming, food supply, and freshwater resources [19,20].

Major water contaminants in Pakistan

Distribution of Major Water Contaminants

Water contamination is not evenly distributed across Pakistan. Studies from different parts of the country have reported arsenic and lead in drinking-water sources, with concentrations varying considerably between locations. Both natural geology and human activities, including industrial discharge and the use of agricultural chemicals, can affect groundwater quality [21,22].

A 2024 study of 79 groundwater samples from the Vehari and Lodhran districts found an average arsenic concentration of 7.7 µg/L and a maximum of 41.4 µg/L. About 33% of the samples exceeded the WHO guideline of 10 µg/L [21]. Arsenic levels above recommended limits have also been reported at several sampling sites in Lahore, showing that the problem affects urban as well as rural areas [22].

Lead contamination was also reported in five districts of Khyber Pakhtunkhwa. Mean concentrations exceeded the WHO limit in every district studied, with Bannu recording the highest average and maximum concentrations [23].

These findings show that water contamination varies from one region to another. Arsenic was detected at concerning levels in the Punjab locations studied, while high lead concentrations were reported across the five sampled districts of Khyber Pakhtunkhwa. This variation supports the need for monitoring and treatment strategies based on local conditions [21-23].

Lead Exposure 

Lead exposure continues to be a public-health concern in Pakistan. Earlier studies identified leaded petrol, work in lead-related industries, and lead-based paint as common sources of childhood exposure [12]. Paint remains an important concern: a 2023 study found that 40% of the residential paints tested contained lead above Pakistan’s legal limit [24]. Lead has also been found in drinking water across several districts of Khyber Pakhtunkhwa [23].

Evidence of contaminated drinking water has been reported in other parts of the country as well. A study in Karachi found an average lead concentration of 146 ppb in groundwater, which is more than 14 times the WHO guideline of 10 ppb [25]. Recent testing in Islamabad also found elevated lead levels, particularly in water supplied through metal pipes. The researchers identified pipe corrosion as an important source of the contamination [26].

Arsenic Exposure 

Arsenic contamination is a serious concern in parts of Punjab. Research from Bahawalpur indicates that arsenic in groundwater comes from both natural geological processes and human activities, including mining, industrial effluents, and agricultural pesticide use [27].

Arsenic contamination levels differ across Pakistan. In rural communities of Kasur and other parts of Punjab, approximately 91% of the groundwater sources tested contained arsenic above recommended levels. Concentrations between 58 and 3,800 µg/L were reported in the villages of Badarpur and Ibrahimabad [28]. Elevated arsenic was also found in water from filtration plants in Islamabad, where the authors identified arsenic in the aquifer and local geohydrological conditions as likely contributors [29].

Other Heavy Metals

Arsenic and lead are not the only metals affecting water quality in Pakistan. Testing conducted after the 2022 floods found chromium, nickel, and lead above recommended limits in groundwater from Charsadda and Nowshera. Cadmium, lead, and nickel were also detected at concerning levels in agricultural soils and sediments [30]. Although the study did not include measurements from before the floods, its findings raise concerns that flooding may redistribute existing contamination between soil, sediment, and nearby water sources. Continued monitoring is needed to determine how long this contamination persists and whether it creates additional exposure risks for affected communities.

Socio-environmental determinants of exposure and vulnerability

Education

Education is a key socio-environmental determinant influencing children's exposure and vulnerability to contaminated water. Caregivers with greater health literacy are more likely to recognize unsafe water sources, adopt appropriate hygiene practices, practice exclusive breastfeeding, and seek timely medical care for diarrheal illness, thereby reducing children's risk of adverse health outcomes [31-33].

Educational disparities remain substantial in Pakistan. A 2023 child development study reported that between 42% and 89% of children under five, depending on the province, have mothers who did not attend or complete primary school [34]. Limited maternal education has consistently been associated with reduced awareness of waterborne disease prevention and delayed healthcare-seeking behaviors [31-33].

Evidence suggests that improving education can reduce children's vulnerability to contaminated water. Evaluations of the Aga Khan Water and Sanitation Extension Programme (WASEP) demonstrated that 94% of rural and 83% of urban water samples from WASEP-supported systems met WHO drinking-water standards, compared with only 8% and 0%, respectively, in control communities [35]. Similarly, a school-based intervention in Karachi improved children's hygiene knowledge and practices [36]. In conclusion, these findings indicate that education strengthens children's protection from unsafe drinking water by improving health knowledge and promoting behaviors that reduce exposure.

Sanitation

Sanitation is a major socio-environmental determinant influencing children's exposure to contaminated water. Inadequate sanitation facilitates fecal contamination of drinking-water sources and increases transmission of pathogens through the fecal-oral route [37].

Although sanitation has improved nationally, important disparities remain. Open defecation has become uncommon in urban areas but continues to affect approximately 12% of the rural population, representing nearly 15 million people [38].

Household hygiene infrastructure has also improved. According to the WHO/UNICEF Joint Monitoring Programme, access to handwashing facilities with soap and water increased substantially between 2017 and 2020 in both rural and urban households [39]. Nevertheless, improved infrastructure has not consistently translated into improved hygiene behaviors. A 2021 national study reported that only 51% of Pakistanis regularly practiced handwashing at critical times, suggesting that behavioral barriers continue to limit the effectiveness of sanitation improvements [7,40].

Collectively, these findings show that sanitation influences children's vulnerability through both infrastructure and behavior. Although sanitation coverage has improved nationally, persistent rural disparities and inconsistent hygiene practices continue to increase children's exposure to contaminated water.

Government Response

Government capacity is a critical socio-environmental determinant influencing children's exposure to unsafe drinking water through water-resource management, sanitation infrastructure, regulatory enforcement, and disaster preparedness. Although Pakistan has established several national policies aimed at improving water security, implementation continues to be a limitation.

The National Water Policy (2018) provides a comprehensive framework for integrated water-resource management across agricultural, domestic, and industrial sectors [40]. However, implementation has progressed slowly because of weak enforcement, overlapping responsibilities between federal and provincial governments, and limited institutional capacity [41-43]. Consequently, many structural contributors to unsafe drinking water remain inadequately addressed.

Climate-related disasters further illustrate the importance of effective governance. The National Disaster Management Plan (NDMP-25) emphasizes anticipatory planning, early warning systems, disaster preparedness, and community resilience [44]. Despite these efforts, communities continue to report delayed warnings, inconsistent emergency response, and fragmented coordination among federal, provincial, and local authorities, limiting the effectiveness of disaster preparedness initiatives [45].

These findings indicate that governance influences children's vulnerability not only through policy development but also through the capacity to implement, coordinate, and sustain effective water-management and disaster-response systems.

Child health outcomes

Waterborne Diseases

Unsafe drinking water exposes children to numerous waterborne pathogens. Contaminated water can transmit bacterial, viral, protozoal, and helminthic organisms that cause diarrhea and other infectious diseases [46]. According to the WHO, diarrheal disease is the third-leading cause of death among children aged 1-59 months and kills approximately 443,832 children under five globally each year [47]. UNICEF Pakistan reports that approximately 53,000 Pakistani children under five die annually from diarrhea associated with inadequate water and sanitation [48].

Transmission commonly occurs through the fecal-oral route, when pathogens from human or animal waste contaminate water or food. Inadequate sanitation and poor hygiene further facilitate the transmission of these pathogens [46,47]. Children in Pakistan face exposure to these conditions, as one study found that 57.5% of children under five were at high risk from inadequate water and sanitation [6].

Flooding can further increase exposure by damaging water and sanitation infrastructure and contaminating drinking-water systems. Following the 2010 floods in northern Pakistan, water samples collected from four locations within a damaged community water-supply system contained *Escherichia coli*, *Shigella*, *Salmonella*, and *Staphylococcus aureus *[49]. 

The consequences extend beyond a single episode of illness. Waterborne pathogens can cause diarrhea, dysentery, typhoid fever, hepatitis A and E, and parasitic infections [46]. Among children in Pakistan, inadequate water and sanitation are also associated with recurrent diarrhea and impaired physical growth, including stunting [6].

Malnutrition and Growth Impairment

Repeated exposure to unsafe water and sanitation conditions can contribute to recurrent diarrheal illness and impaired childhood growth. In Pakistan, inadequate water and sanitation are associated with increased risks of diarrhea and stunting among children under five [6].

The 2022 floods intensified Pakistan’s existing nutrition crisis. Beginning in September 2022, health professionals screened more than 22,000 children at facilities in flood-affected areas of Sindh and Balochistan and diagnosed over 2,630 (approximately one in nine) with severe acute malnutrition [50]. The 2025 Joint Child Malnutrition Estimates also document the continuing burden of childhood stunting in Pakistan [51]. Food insecurity is additionally shaped by broader socioeconomic conditions, including unemployment, population growth, corruption, and economic instability [52].

Prenatal chemical exposure may further affect early growth. In a prospective cohort study conducted in China, higher maternal urinary arsenic concentrations during the third trimester were associated with greater odds of infants being born small for gestational age. Among male infants, maternal arsenic concentrations were also negatively associated with weight and length at six and twelve months [53].

Lead Exposure

Lead is a well-established neurotoxicant that can adversely affect children’s intellectual development. Elevated blood lead concentrations have been associated with lower IQ scores, behavioral problems, and diminished school performance [54]. In an international pooled analysis, an increase in concurrent blood lead concentration from 2.4 to 30 µg/dL was associated with an estimated 6.9-point reduction in IQ (95% CI: 4.2-9.4) [54]. The association was evident even at concentrations below 7.5 µg/dL, and the investigators found no clear threshold below which lead exposure was not associated with intellectual deficits [54]. Elevated blood lead concentrations have also been documented among children in Pakistan, although evidence concerning their specific health effects within Pakistan remains limited [12].

Arsenic Exposure

Evidence indicates that arsenic exposure may adversely affect children’s neurodevelopment. Among school-aged children in Lahore and Kasur, combined exposure to arsenic and fluoride through drinking water was associated with lower IQ scores and greater oxidative stress [10]. A broader review of studies conducted in Asia also identified associations between early-life arsenic exposure, neurocognitive impairment, and epigenetic changes [55].

Long-term inorganic arsenic exposure has been associated with cardiovascular disease, infant mortality, adverse pregnancy outcomes, and cancers of the skin, bladder, and lungs [56]. A nationwide Danish cohort study also found that maternal exposure to arsenic in drinking water was associated with an increased risk of congenital heart disease in offspring [57].

In Pakistan, an investigation of groundwater in the Pind Dadan Khan area of the Jhelum Basin found arsenic concentrations ranging from 0.5 to 100 µg/L. The study’s health-risk assessment produced hazard quotient values above 1, indicating the potential for chronic non-carcinogenic health effects [58].

Intervention strategies and policy implications

Strengthening Water Governance

Effective governance is central to improving Pakistan’s water security. The country has adopted national frameworks including the National Water Policy 2018 and the NDMP 2025 [40,44]. However, analyses of Pakistan’s water sector have identified unclear institutional responsibilities, weak administrative capacity, limited policy continuity, and persistent barriers to implementation [41-43]. Strengthening institutional accountability, clarifying the responsibilities of federal and provincial authorities, and expanding implementation capacity may help translate these policies into improvements in water management and, indirectly, public health.

Recent initiatives demonstrate progress toward these objectives. The Water Resource Accountability in Pakistan (WRAP) program includes a component implemented by the International Water Management Institute to strengthen water-management capacity at the federal, provincial, and district levels. It also supports implementation of the Punjab Water Act 2019 and the Khyber Pakhtunkhwa Water Act 2020 [59]. Continued support for these initiatives may contribute to more accountable and sustainable management of Pakistan’s water resources.

Sustainable Water Resource Management

Protecting drinking-water quality requires addressing the environmental processes that contribute to contamination. Unregulated groundwater extraction has lowered water tables, increased pumping costs, degraded groundwater quality, and intensified soil-salinity problems in irrigated areas, threatening the long-term sustainability of agriculture [20].

The literature supports stronger groundwater regulation, improved irrigation efficiency, rehabilitation of water infrastructure, and strategic water planning [20,60]. Pakistan-specific research has also identified crops and plant species that tolerate saline conditions [61]. Although tube wells provide access to groundwater, expanding extraction without effective regulation can accelerate aquifer depletion and water-quality deterioration [20]. Preserving water quality is important for public health because unsafe drinking water and chemical contamination contribute to disease, while safely managed drinking-water services can reduce associated health risks [62].

Community-Based Public Health Interventions

Addressing environmental contamination alone is unlikely to eliminate children's exposure to unsafe drinking water. Throughout this review, education, sanitation, and hygiene consistently emerged as major determinants of exposure and vulnerability, highlighting the importance of community-based public health interventions.

WASH programs have demonstrated measurable improvements in both water quality and childhood health outcomes. Evaluations of the Aga Khan WASEP found substantially lower diarrheal disease transmission and greater compliance with WHO drinking-water standards in intervention communities than in comparison villages [35,63]. Similarly, sanitation initiatives such as the Orangi Pilot Project and Community-Led Total Sanitation (CLTS) have expanded household sanitation infrastructure, reduced open defecation, and promoted community participation in hygiene improvement efforts [64].

Despite these advances, persistent rural disparities and inconsistent hygiene behaviors indicate that infrastructure improvements alone are insufficient. Continued investment in community education, hygiene promotion, sanitation infrastructure, and sustained behavior-change programs will be necessary to maximize the long-term effectiveness of WASH interventions [7,39].

Limitations and future directions

This review has several limitations. As a narrative review, it does not include a formal systematic review protocol, quantitative meta-analysis, or formal risk-of-bias assessment. Although a structured literature search was conducted using multiple databases and reports from major international organizations, relevant studies may have been missed. In addition, much of the available evidence consists of regional or cross-sectional studies, limiting the ability to establish causal relationships and reducing the generalizability of findings across Pakistan.

Future research should prioritize nationally representative longitudinal studies to better characterize the relationships among environmental determinants, socio-environmental determinants, water contamination, and child health outcomes. Additional evaluation of intervention effectiveness, including WASH programs, sustainable groundwater management strategies, and water governance initiatives, is also needed to identify evidence-based approaches for reducing childhood exposure to contaminated drinking water and improving long-term health outcomes.

## Conclusions

This narrative review demonstrates that Pakistan's water crisis cannot be attributed to a single environmental hazard but instead reflects complex interactions among environmental determinants, major water contaminants, and socio-environmental determinants that influence children's exposure, vulnerability, and health outcomes.

The evidence synthesized throughout this review indicates that addressing only one aspect of Pakistan's water crisis is unlikely to produce sustainable improvements in child health. Rather, effective solutions require coordinated strategies that simultaneously reduce environmental contamination, improve sanitation and hygiene, expand public education, and promote sustainable water-resource management. Protecting children from unsafe drinking water is therefore not solely an environmental or public health priority but a long-term investment for Pakistan. Continued implementation of existing evidence-based interventions will be essential to reduce childhood exposure to contaminated water and improve health outcomes for future generations.

## References

[1] World Health Organization (2017). Guidelines for drinking-water quality, 4th edition, incorporating the 1st addendum. World Health Organization.

[2] Kuzma S, Saccoccia L, Chertock M (2026). 25 countries, housing one-quarter of the population, face extremely high water stress. World Resources Institute.

[3] Ahmad S, Zahra J, Ali M (2022). Impact of water insecurity amidst endemic and pandemic in Pakistan: two tales unsolved. Ann Med Surg (Lond).

[4] Kumar L, Kumari R, Kumar A (2023). Water quality assessment and monitoring in Pakistan: a comprehensive review. Sustainability.

[5] Ishaque W, Mukhtar M, Tanvir R (2023). Pakistan’s water resource management: ensuring water security for sustainable development. Front Environ Sci.

[6] Murtaza F, Muzaffar M, Mustafa T, Anwer J (2021). Water and sanitation risk exposure in children under-five in Pakistan. J Family Community Med.

[7] World Health Organization, United Nations Children’s Fund (2023). Progress on household drinking water, sanitation and hygiene 2000-2022: special focus on gender. https://data.unicef.org/resources/jmp-report-2023/.

[8] Fatima M, Ünsal Ö (2026). Geospatial analysis of diarrhoea determinants among children under five in Pakistan using multiscale geographically weighted regression (MGWR). Appl Geogr.

[9] Rahmat ZS, Zubair A, Abdi I, Humayun N, Arshad F, Essar MY (2026). The rise of diarrheal illnesses in the children of Pakistan amidst COVID-19: a narrative review. Health Sci Rep.

[10] Saeed M, Rehman MY, Farooqi A, Malik RN (2022). Arsenic and fluoride co-exposure through drinking water and their impacts on intelligence and oxidative stress among rural school-aged children of Lahore and Kasur districts, Pakistan. Environ Geochem Health.

[11] Heidari S, Mostafaei S, Razazian N, Rajati M, Saeedi A, Rajati F (2022). The effect of lead exposure on IQ test scores in children under 12 years: a systematic review and meta-analysis of case-control studies. Syst Rev.

[12] Kadir MM, Janjua NZ, Kristensen S, Fatmi Z, Sathiakumar N (2008). Status of children's blood lead levels in Pakistan: implications for research and policy. Public Health.

[13] Caretta MA, Mukherji A, Arfanuzzaman M (2022). Water. Climate Change 2022: Impacts, Adaptation and Vulnerability.

[14] (2026). More than 10 million people, including children, living in Pakistan’s flood-affected areas still lack access to safe drinking water. https://www.unicef.org/press-releases/more-10-million-people-including-children-living-pakistans-flood-affected-areas.

[15] Campbell-Lendrum D, Corvalán C (2007). Climate change and developing-country cities: implications for environmental health and equity. J Urban Health.

[16] Somani R (2023). Global warming in Pakistan and its impact on public health as viewed through a health equity lens. Int J Soc Determinants Health Health Serv.

[17] World Weather Attribution (2026). Climate change intensified heavy monsoon rain in Pakistan, exacerbating urban floods that impacted highly exposed communities. https://www.worldweatherattribution.org/climate-change-likely-intensified-heavy-monsoon-rain-in-pakistan-exacerbating-urban-floods-that-impacted-highly-exposed-communities/.

[18] (2026). UNICEF statement on monsoon floods’ impact on children in Pakistan. https://www.unicef.org/asia-pacific/press-releases/unicef-statement-monsoon-floods-impact-children-pakistan.

[19] Ishaque W, Tanvir R, Mukhtar M (2022). Climate change and water crises in Pakistan: implications on water quality and health risks. J Environ Public Health.

[20] Qureshi AS (2020). Groundwater governance in Pakistan: from colossal development to neglected management. Water.

[21] Khalid S, Shahid M, Bibi I (2024). Assessing the arsenic contents and associated risks in groundwater of Vehari and Lodhran districts, Pakistan. Water.

[22] Nawaz R, Nasim I, Irfan A (2023). Water quality index and human health risk assessment of drinking water in selected urban areas of a mega city. Toxics.

[23] Shakoor H, Ahmad F, Shahab M (2025). Vulnerability and risk assessment of lead (Pb) concentrations in drinking water via statistical and geostatistical analyses. Frontiers in Water.

[24] Siddiqui D, Coulter L, Loudon C (2023). 'The brighter the worse': lead content of commercially available solvent-based paints intended for residential use in Pakistan. F1000Research.

[25] Ul-Haq N, Arain MA, Badar N, Rasheed M, Haque Z (2011). Drinking water: a major source of lead exposure in Karachi, Pakistan. East Mediterr Health J.

[26] Waseem K, Akhtar AS, Nawaz A (2025). Assessment of lead contamination in drinking water in zone I and zone III of Islamabad, Pakistan. Discov Water.

[27] Ullah Z, Zeng XC, Rashid A (2023). Integrated approach to hydrogeochemical appraisal of groundwater quality concerning arsenic contamination and its suitability analysis for drinking purposes using water quality index. Sci Rep.

[28] Arshad N, Imran S (2017). Assessment of arsenic, fluoride, bacteria, and other contaminants in drinking water sources for rural communities of Kasur and other districts in Punjab, Pakistan. Environ Sci Pollut Res Int.

[29] Sohail M, Ehsan M, Riaz S (2022). Investigating the drinking water quality and associated health risks in metropolis area of Pakistan. Front Mater.

[30] Gul N, Khan N, Mehmood N (2025). A comprehensive assessment of heavy metal pollution in flood-affected areas in Pakistan: a case study of the 2022 superflood through pollution and water quality indices. Sustainable Water Resources Management.

[31] Arif S, Khan H, Aslam M, Farooq M (2021). Factors influencing exclusive breastfeeding duration in Pakistan: a population-based cross-sectional study. BMC Public Health.

[32] Ganbold G, Farnaz N, Scutts T, Borg B, Mihrshahi S (2025). The association between exclusive breastfeeding and diarrhoea morbidity in infants aged 0-6 months: a rapid review and meta-analysis. Matern Child Nutr.

[33] Das JK, Siddiqui F, Padhani ZA (2023). Health behaviors and care seeking practices for childhood diarrhea and pneumonia in a rural district of Pakistan: a qualitative study. PLoS One.

[34] Hentschel E, Tomlinson H, Hasan A, Yousafzai A, Ansari A, Tahir-Chowdhry M, Zamand M (2023). Risks to child development and school readiness among children under six in Pakistan: findings from a nationally representative phone survey. Int J Early Child.

[35] Shah A, Ali M, Ali K (2022). Assessing water and water infrastructure quality in community-managed water supply systems in northern Pakistan. Water Practice and Technology.

[36] Pradhan NA, Hashmi M, Mazhar L (2025). Intervention to improve children's hygiene in urban squatter settlement schools in Pakistan: an implementation research. Environ Health Insights.

[37] United Nations Children’s Fund (2018). Offering solutions: an appraisal of WASH sustainability challenges in Pakistan (WASH Field Note FN/10/2018). https://www.unicef.org/rosa/media/11811/file.

[38] WHO and UNICEF (2021). Progress on Household Drinking Water, Sanitation and Hygiene: 2000-2020 - Five Years into the SDGs. https://washdata.org/sites/default/files/2021-07/jmp-2021-wash-households.pdf.

[39] UN-Water UN-Water (2026). SDG 6 country acceleration case studies 2022: Pakistan. Pakistan: SDG 6 country acceleration case studies.

[40] Asian Development Bank (2026). National water policy, 2018 (Pakistan). https://lpr.adb.org/resource/national-water-policy-2018-pakistan.

[41] Sumra K, Mumtaz M, Khan K (2020). National water policy of Pakistan: a critical analysis. J Manag Sci.

[42] Aisha N, Ghani F, Fatima N (2026). Water Governance in Pakistan for Climateinclusive Irrigation Water Management and Food Security. https://www.adpc.net/igo/category/ID1814/doc/2022-f30Md5-ADPC-Water_Governance_in_Pakistan.pdf.

[43] Stimson Center. (2023, November 27 (2026). Pakistan’s political economy perpetuates its water crisis. https://www.stimson.org/2023/pakistans-political-economy-perpetuates-its-water-crisis/.

[44] National Disaster Management Authority. (2025 (2026). National disaster management plan 2025 (NDMP-2025). https://www.ndma.gov.pk/storage/plans/March2025/sps3g997GvbYIOEpwvKw.pdf.

[45] Shah A, Ullah A, Khan N (2023). Community social barriers to non-technical aspects of flood early warning systems and NGO-led interventions: the case of Pakistan. Frontiers in Earth Science.

[46] Ramírez-Castillo FY, Loera-Muro A, Jacques M, Garneau P, Avelar-González FJ, Harel J, Guerrero-Barrera AL (2015). Waterborne pathogens: detection methods and challenges. Pathogens.

[47] (2026). Diarrhoeal disease. https://www.who.int/news-room/fact-sheets/detail/diarrhoeal-disease.

[48] (2026). WASH: water, sanitation and hygiene. https://www.unicef.org/pakistan/wash-water-sanitation-and-hygiene-0.

[49] Baig SA, Xu X, Khan R (2012). Microbial water quality risks to public health: potable water assessment for a flood-affected town in northern Pakistan. Rural Remote Health.

[50] Ahmed T, Zounemat-Kermani M, Scholz M (2026). More than 1 in 9 children in flood-affected areas of Pakistan suffering from severe acute malnutrition. Int. J. Environ.

[51] (2025). Levels and trends in child malnutrition: UNICEF/WHO/World Bank Group joint child malnutrition estimates: key findings of the 2025 edition. https://www.who.int/publications/i/item/9789240112308.

[52] Habib S, Didar H (2025). Analyzing the composite effect of corruption and socio-economic variables on food insecurity in Pakistan: a comprehensive study. Population and Economics.

[53] Cai P, He H, Song X, Qiu T, Chen D, Zhang H (2024). Association between gestational arsenic exposure and infant physical development: a prospective cohort study. BMC Public Health.

[54] Lanphear BP, Hornung R, Khoury J (2005). Low-level environmental lead exposure and children's intellectual function: an international pooled analysis. Environ Health Perspect.

[55] Hu K, Islam MA, Parvez F, Bhattacharya P, Khan KM (2024). Chronic exposure of arsenic among children in Asia: a current opinion based on epidemiological evidence. Curr Opin Environ Sci Health.

[56] (2025). Arsenic. World Health Organization.

[57] Richter F, Kloster S, Wodschow K (2022). Maternal exposure to arsenic in drinking water and risk of congenital heart disease in the offspring. Environ Int.

[58] Ullah Z, Rashid A, Ghani J, Talib MA, Shahab A, Lun L (2023). Arsenic contamination, water toxicity, source apportionment, and potential health risk in groundwater of Jhelum Basin, Punjab, Pakistan. Biol Trace Elem Res.

[59] International Water Management Institute (2026). Water resource accountability in Pakistan (WRAP). https://www.iwmi.org/projects/wrap/.

[60] Ali M, Akram MM, Silverman E (2024). Sustainable water solutions for agriculture in Pakistan: an overview. J Innov Sci.

[61] Heuperman AF, Kapoor AS, Denecke HW (2002). Biodrainage: Principles, Experiences and Applications. https://www.fao.org/4/y3796e/y3796e00.htm.

[62] (2026). Water, sanitation and hygiene (WASH). https://www.who.int/health-topics/water-sanitation-and-hygiene-wash.

[63] Nanan D, White F, Azam I, Afsar H, Hozhabri S (2003). Evaluation of a water, sanitation, and hygiene education intervention on diarrhoea in northern Pakistan. Bull World Health Organ.

[64] Orangi Pilot Project Research and Training Institute (1996). The Orangi Pilot Project, Pakistan. https://www.iied.org/7037iied.

